# Graphene Oxide Sheets Combine into Conductive Coatings by Direct Oxidative Electropolymerization

**DOI:** 10.1038/s41598-017-05269-1

**Published:** 2017-07-10

**Authors:** S. Halevy, Y. Bochlin, Y. Kadosh, A. Kaplan, H. Avraham, A. Nissim, R. Ben Hamo, T. Ohaion-Raz, E. Korin, A. Bettelheim

**Affiliations:** 1 Chemical Engineering Department, Ben-Gurion University of the Negev, Beer-Sheva, 84105 Israel; 2 Chemistry Department, NRCN, P.O. Box 9001, Beer-Sheva, 84190 Israel; 3 Chemical Engineering Department, MIT, Cambridge, MA 02139 USA

## Abstract

New coatings are obtained when graphene oxide is further oxidized at moderate anodic potentials (≤~1.3 V vs. Ag/AgCl). Based on a variety of spectroscopic and electrochemical observations, the coatings are attributed to the direct electropolymerization of graphene oxide sheets via oxidation of the phenol edge groups on graphene. Depending on the applied potential, ether or carboxylic groups are formed. The coatings obtained via further oxidation are characterized by a lower O/C ratio due to decarboxylation and a higher content of C=C bonds. These bonds extend aromatic conjugation into the combined graphene oxide sheets and are responsible for the highly conductive nature of these coatings.

## Introduction

Graphene has generated increased interest in fundamental science and its potential applications due to its unique structure and properties, such as its excellent conductivity, high electron mobility, superior chemical stability, large surface-to-volume ratio^1^, and high transparency^2^. The surface properties of graphene can also be adjusted via chemical modifications, and this offers opportunities for the preparation and possible application of functionalized materials^3^. Such applications have been demonstrated in a variety of fields, such as energy storage^4^, catalysis^5^, and biosensing^6^. Recent comprehensive reviews concerning the properties of graphene derivatives and their applications, including in the field of energy conversion, are available^7–10^.

Different approaches to synthesize graphene and graphene nanostructures have been developed, such as direct chemical synthesis^11, 12^, chemical vapor deposition (CVD) of hydrocarbons on metal substrates^13^, epitaxial growth on single-crystal metals^14^ exfoliation from bulk graphite^15^, and tailoring graphene sheets or carbon nanotubes into nanoribbons^16, 17^.

Chemically converted graphene suspensions are versatile and have permitted the use of a large number of deposition techniques, such as spray coating^7^. These techniques have been used to produce films with coverages ranging from evenly spaced single sheets to densely packed, overlapping films. Graphene films on electrodes are usually obtained by drop-casting a graphene suspension obtained via the chemical reduction of graphene oxide (GO) sheets^18^. This preparation methodology has limitations, such as a lack of control of the film thickness, which can be circumvented using electrochemical techniques. Recently, the electrochemical reduction of GO to graphene has drawn attention due to its fast and green nature^19–21^. The synthesis of high-quality graphene nanosheets via the electrochemical reduction of chemically exfoliated GO has been reported^19^, and conductively reduced graphene oxide (rGO) can be precipitated on an electrode surface at cathodic potentials (≤−1 V) due to its reduced solubility compared to GO^19, 22^. The present paper shows that, surprisingly, conductive coatings are obtained when GO is further oxidized at moderate anodic potentials (≤~1.3 V vs. Ag/AgCl). In contrast to reports concerning the introduction of GO or rGO in conducting polymer films obtained via the electropoloymerization (EP) of monomers, such as pyrrole or thiophene derivatives^23, 24^, the phenomenon in the present case is attributed to the direct EP of GO sheets via oxidation of the phenol edge groups. A general scheme for the formation and unexpected conductive nature of the coatings is presented based on microscopic (SEM, TEM, AFM), spectroscopic (FTIR, Raman, XPS) and electrochemical (cyclic voltammetry - CV, electrochemical quartz crystal microbalance - EQCM) observations.

## Results and Discussion

**Figure 1:**
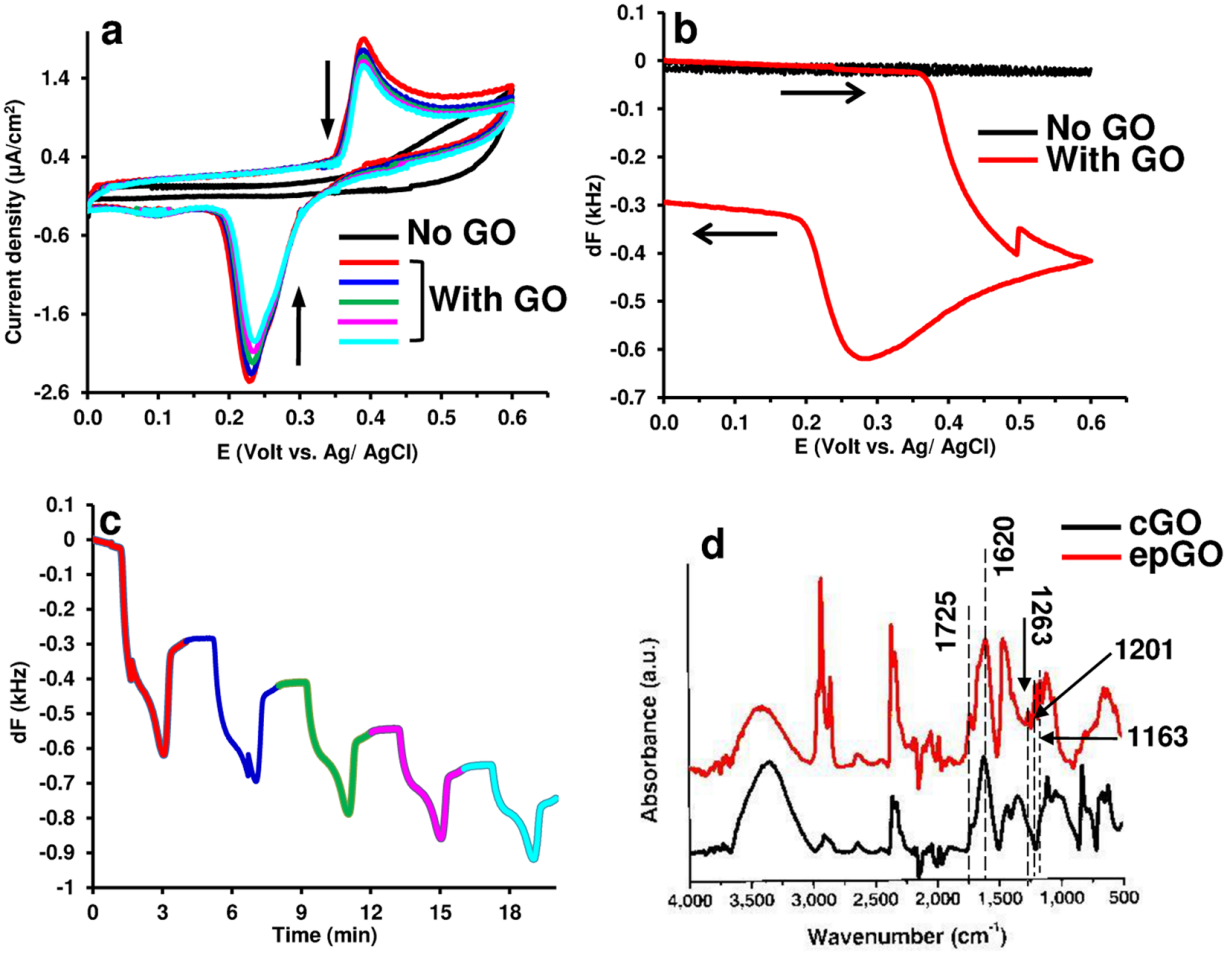
Following the growth of the EP coatings using CV and EQCM and their characterization via FTIR compared to cGO. (**a**) CVs (25 mv/s) for the Au-coated quartz crystal electrode in 0.1 M NaHCO_3_ in the absence and presence of 2 mg/mL GO (current decreases according to the arrows in consecutive scans). (**b**) EQCM frequency response in the absence and presence of GO. (**c**) Frequency change for five consecutive scans in the presence of GO. (**d**) FTIR spectra of the cGO and epGO coating obtained as described in (**c**).

The CVs obtained by cycling the potential of an Au- coated quartz crystal electrode in the range from 0 to 0.6 V vs. Ag/AgCl in a GO-NaHCO_3_ emulsion solution at pH 8.3 are shown in Fig. 1a. A quasi-reversible redox couple was observed, and its half-wave potential (E_1/2_) of 0.31 V agrees with the reported peak potentials for the one electron oxidation of phenol and some of its derivatives^25^ when a 59 mV/pH dependence is considered. The redox couple is undetectable at higher pH values, which seems to be in accordance with the report that the phenolic groups of GO at pH ≥ 9 ionize into phenolate anions that are subsequently converted to ketones^26^. The quartz crystal frequency as a function of the potential obtained in the first potential scan is shown in Fig. 1b. A sharp decrease and increase in the frequency (increase and decrease in the mass, respectively) were observed at potentials of 0.36 and 0.26 V, respectively. The average potential of these changes, 0.31 V, coincides with the E_1/2_ of the electrode reaction, and the overall frequency decrease for the whole potential scan is almost twice that of the overall increase. This finding indicates that the oxidation of the GO phenolic groups forms products that are partly insoluble in the electrolyte and subsequently precipitate on the electrode surface, and this phenomenon has also been reported for polymeric films obtained via the electro-oxidation of phenol and its derivatives^27^. Continuous potential scanning causes a gradual decrease in the peak currents (Fig. 1a), while the mass accumulating on the electrode surface results in an overall increase over the 5 potential scans, as shown in Fig. 1c. The FTIR spectrum of the film obtained on the Au-quartz crystal electrode is depicted in Fig. 1d and is compared to spectrum of the cast GO (cGO). It has been recognized that the spectra of GO samples are difficult to interpret due to overlapping bands that come from the many chemical bonds^28^. The two spectra do not differ significantly in the 3700–2000 cm^−1^ range, which contains the peaks of the O-H (~3300 cm^−1^), C-H (2855, 2927 cm^−1^), and CO_2_ (2355 cm^−1^) bonds. The two spectra also have a band at approximately 1725 cm^−1^, which was assigned to a carbonyl group, and one at ~1620 cm^−1^, which has been attributed to C=C bonds in some reports but is usually assigned to water bending modes^28–30^. The appearance of new peaks at 1163, 1201, and 1263 cm^−1^ is indicative of the formation of new C-O-C ether bonds^31^ in the electrochemically prepared films. These bonds, which can form between phenolic groups on different GO sheets, are probably responsible for combining the sheets.

**Figure 2:**
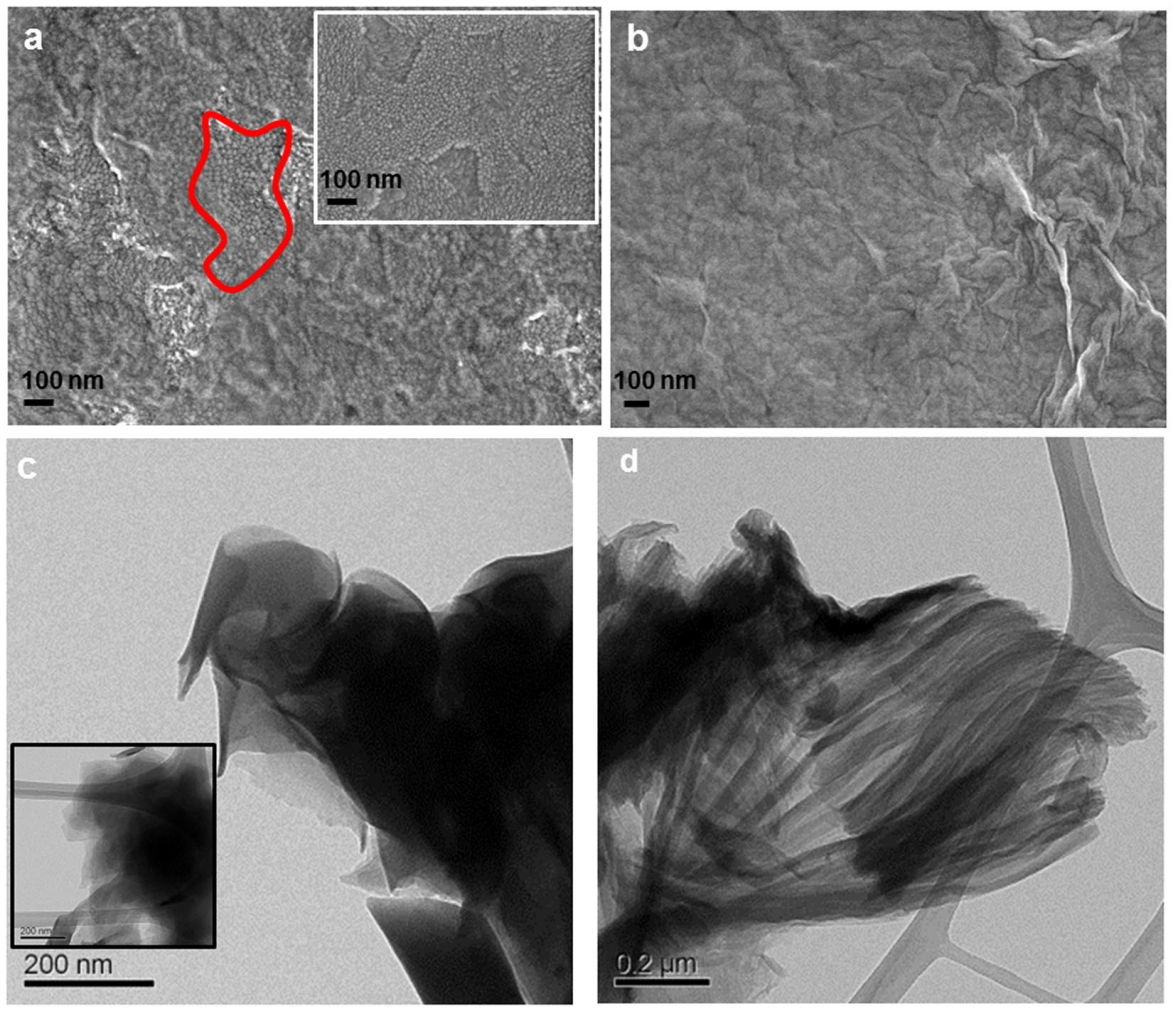
SEM and TEM images of the GO coatings obtained via casting and EP. SEM top view images for (**a**) ITO/epGO_0.8_ (grains found in bare ITO shown in the inset are observed in the region marked in red) and (**b**) ITO/epGO_1.3_. TEM micrographs for (**c**) ITO/epGO_0.8_ (inset: ITO/cGO) and (**d**) ITO/epGO_1.3_.

Further characterization of the coatings obtained via GO-NaHCO_3_ emulsions was achieved using ITO (indium-tin oxide) and GC (glassy carbon) electrodes, which were used because of their lower oxidation tendency and higher electrochemical window anodic potential limit (~1.1 compared to ~0.8 V for Au at pH 8.3). The film growth on ITO, as followed by CV in the potential range 0 to 1.3 V (see Supplementary Fig. S1b), showed similar features to those obtained on Au (Fig. 1a). The peak currents for the phenolic group redox reaction (not observed for the buffer solution devoid of GO, shown in Supplementary Fig. S1a) gradually decrease during continuous potential scanning. Chronoamperometry (CA), which was conducted at 0.8 and 1.3 V for a period of 0.5 h, resulted in steady state current densities of 2 and 6.5 µA/cm^2^, respectively (see Supplementary Fig. S1c). The SEM surface images for the resulting coatings, considered to be obtained via the direct EP of GO sheets and abbreviated epGO_0.8_ and epGO_1.3_, respectively, are shown in Fig. 2a and b and were compared to the image for bare ITO (inset in Fig. 2a). While the epGO_0.8_ coating obtained at a low potential shows incomplete surface coverage (grains of bare ITO can still be observed in the region marked in red), epGO_1.3_ exhibits full coverage of the surface and is characterized by the presence of more folding and wrinkles. More insight into the morphology of the coatings was obtained using TEM, as demonstrated in Fig. 2c and d for the epGO_0.8_ and epGO_1.3_ coatings, respectively. In contrast to the flat nature of the cGO films (inset of Fig. 2c), the images obtained for epGO_0.8_ show the sheet edges curling (Fig. 2c), which possibly indicates expansion of the sheet dimensions. This feature seems to precede the formation of high-area, fan-like structures, in the epGO_1.3_ sample as demonstrated in Fig. 2d. The epGO_1.3_ films were also thicker than the epGO_0.8_ films, 30 vs. 7 nm and the surface roughnesses were 8 and 3 nm, respectively, as determined via AFM (see Supplementary Fig. S2).

**Figure 3:**
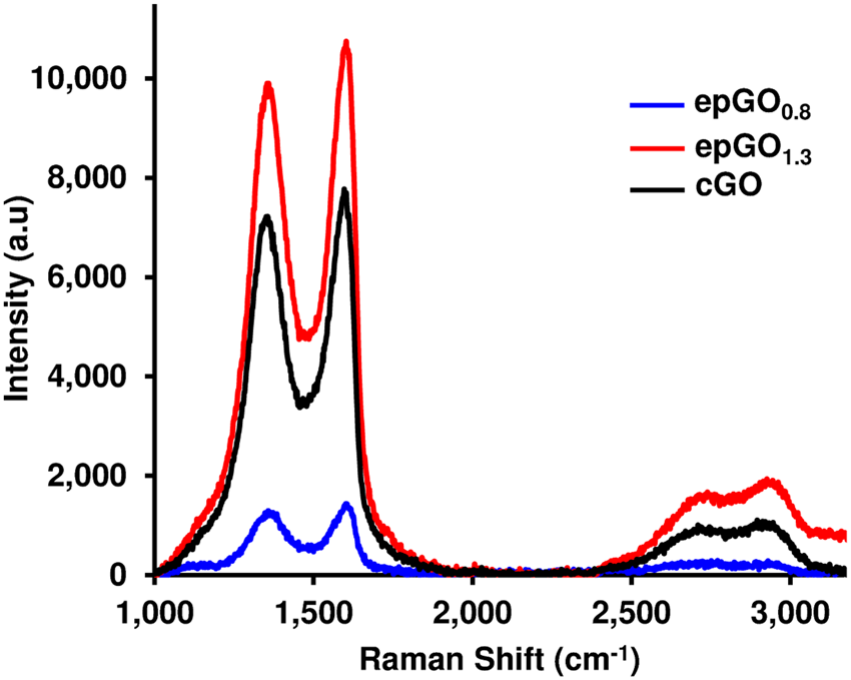
Raman spectroscopy characterization of the EP coatings compared to coatings obtained via casting. Raman spectra obtained after a baseline correction for coatings on ITO of epGO_0.8_, epGO_1.3_, and cGO.

The Raman spectra for the epGO_0.8_ and epGO_1.3_ coatings on ITO were compared to that of cGO and are shown in Fig. 3. All the Raman spectra exhibited two intense peaks: the G-band (~1600 cm^−1^), which is due to the bond stretching of the sp^2^ carbon pairs in both the rings and chains, and the D-band (~1360 cm^−1^), which is related to the breathing mode of the aromatic rings and requires a defect for its activation^32^. The overtone of the D peak, the 2D peak, appears at ~2710 cm^−1^, and its shift and shape have been correlated with the number of graphene layers^33^. The peak near 2920 cm^−1^, the D + D′ peak, is also defect-activated^34^. The shape and position of the D and D + D′ peaks for the epGO coatings do not differ significantly from those of the cGO coating, which indicates that the anodic polarization does not contribute to the massive formation of new defects in the epGO coatings^32, 35^. The intensity ratio of the D and G peaks (I_D_/I_G_) has been used as an indicator of disorder in graphene, i.e., disorder arising from ripples, edges and the presence of domain boundaries^36^. The I_D_/I_G_ values obtained for cGO, epGO_0.8_, and epGO_1.3_ are 0.94, 0.89, and 0.93, respectively. Considering the low accuracy of the I_D_ and I_G_ values for epGO_0.8_, which is a result of the low surface coverage of the film, the I_D_/I_G_ ratio values for the three films are almost identical. This result indicates that the EP process does not necessarily increase the disorder and defect intensity in comparison to the GO coatings obtained via casting. The full width measurements at half maximum of the D peaks (Γ_D_) for the three coatings were 177, 143, and 176 cm^−1^ and those of the G peaks (Γ_G_) were 130, 103, and 118 cm^−1^, respectively. EP, therefore, does not cause broadening of the peaks, which indicates a higher concentration of defects^37^. The data seem to lead to the conclusion that the modifications introduced by the electrochemical oxidation are preferentially located on the edges of the GO sheets^38, 39^.

**Figure 4:**
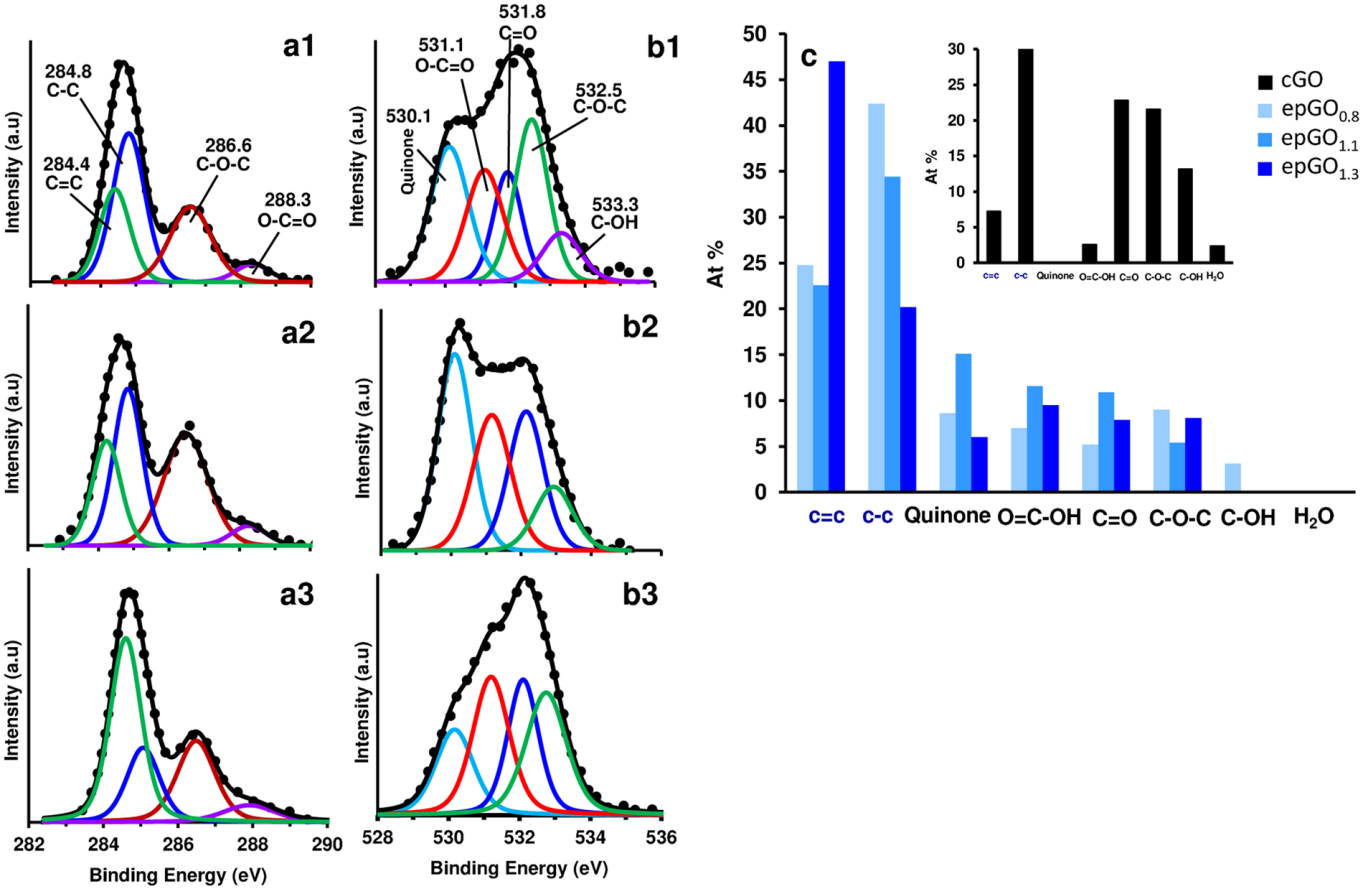
Characterization of coating functionalities via XPS. XPS C1s spectra for epGO_0.8_, epGO_1.1_, and epGO_1.3_ on ITO (**a1**, **a2**, and **a3**, respectively). O1s spectra for epGO_0.8_, epGO_1.1_, and epGO_1.3_ (**b1**, **b2**, and **b3**, respectively). Atomic percentages for the various functional groups in cGO, epGO_0.8_, epGO_1.1_, and epGO_1.3_ (**c**).

The films obtained via CA at 0.8, 1.1, and 1.3 V were examined using XPS to further determine the nature of their functionalities. The C1s spectra for the epGO_0.8_, epGO_1.1_, and epGO_1.3_ films are shown in Fig. 4 a1, a2, and a3, respectively, and the O1s respective spectra are shown in Fig. 4 b1, b2, and b3. The calculated O/C ratios for the three films were 0.61, 0.70, and 0.36, respectively, compared to 0.68, which was obtained from the cGO spectra (see Supplementary Fig. S3). Although the ratios for the films obtained at low potentials show small deviations from that of cGO, a sharp decrease occurred for epGO_1.3_. In contrast to the present case, in which the films were obtained on different electrodes via bulk electrolysis of a GO emulsion at different potentials, it has been reported that the O/C ratio increases as the anodic potential applied to a film of cGO gradually increases^38^. This seems to indicate that a deoxygenation/decarboxylation process is responsible for the decrease in the oxygen content in the films obtained via anodic polarization in the present case. Decarboxylation has also been suggested to occur in the case of electrochemically oxidized carbon fibers^40^, and spontaneous self-deoxygenation has been reported for GO in alkaline solutions via a disproportionation reaction that results in the formation of CO_2_ and the extension of conjugation^28, 39^.

The C1s spectra were fitted with two peaks for hydrocarbons: sp^2^ C=C and sp^3^ C-C (284.4 and 284.8 eV, respectively), and the peaks generally related to the carbon-oxygen species. The calculated C=C/C-C content ratio was 0.24 for cGO, and 0.58, 0.66, and 2.33 for the films obtained via EP at 0.8, 1.1 and 1.3 V, respectively. A more detailed analysis of the C-O functionalities was obtained from the O1s spectra, which were fitted to the peaks for quinone, COOH, C=O, C-O-C, C-OH (530.1, 531.1, 531.8, 532.5, and 533.3 eV, respectively), and adsorbed water (534.0 eV) observed for cGO. Figure 4c shows the concentration profiles for the various functionalities of cGO (inset) as well as the three coatings obtained via EP. Because the EP films were not obtained by further oxidation of cGO, there is not a common base for a comparison of a particular functionality concentration between the two types of coatings. However, the comparison for the EP coatings shows that the phenolic C-OH group appears only in epGO_0.8_ (3%), which clearly indicates complete oxidation at more anodic potentials. The concentrations of the quinone groups as well of those of the C=O (ketone) and carboxyl groups increased when the applied potential increased from 0.8 to 1.1 V and decreased for epGO_1.3_. This result was attributed to the increase in the oxygen functionalities at potentials as high as 1.1 V and the deoxygenation/decarboxylation process that occurs at higher potentials, which was in agreement with the calculated O/C ratios for the three applied potentials. The C-O-C (ether) concentration profiles show a different behavior (increased concentration at 1.3 V), which probably indicates newly formed ether bonds are connecting the epGO sheets and, unlike other oxygen functionalities positioned at the sheet edges, are less prone to further oxidation.

**Figure 5:**
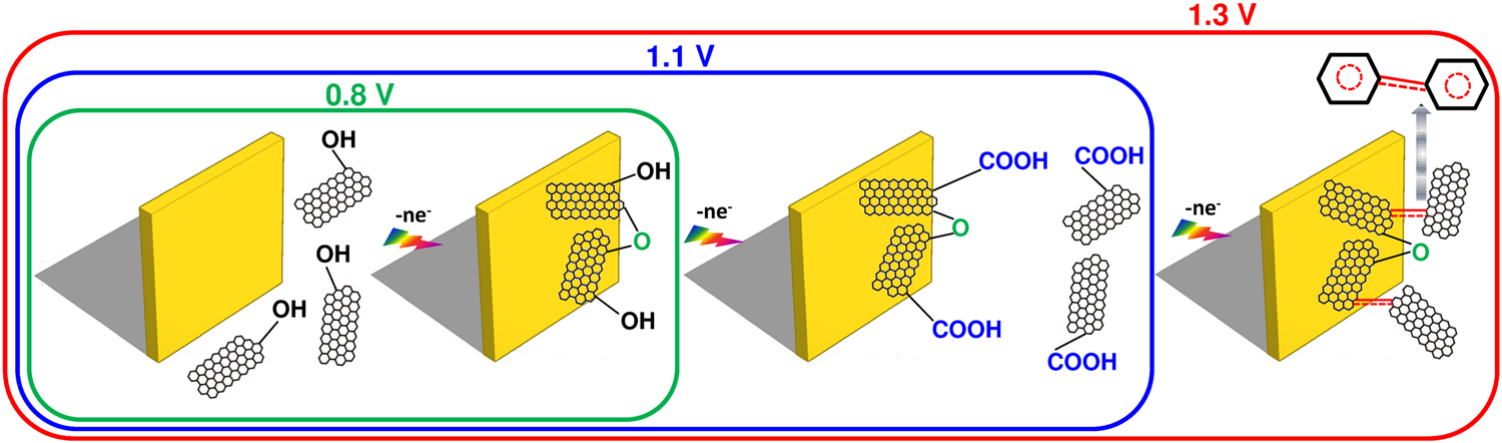
Scheme for the formation of the epGO coatings. The effect of the applied potential on the growth of the coatings obtained via EP.

A general scheme for the formation of the epGO coatings is presented in Fig. 5. The GO sheets combined at low applied potentials (≤0.8 V) through the ether groups formed via the EP of the phenolic edge groups. The higher applied potentials (~1.1 V) form carboxyl-rich coatings that, upon further oxidation at oxygen evolving potentials (~1.3 V), are converted via decarboxylation to coatings with lower O/C ratios and higher C=C content. This finding is also in accordance with reports concerning other electrochemically oxidized carbon-related systems in which decarboxylation^40^ and extension of conjugation through C=C bonds are involved^28^.

**Figure 6:**
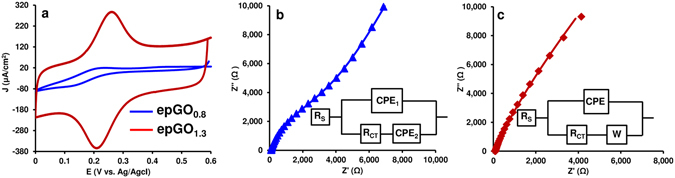
Examining the conductive nature of the EP coatings via CV and EIS. (**a**) CVs at a scan rate of 100 mV/s for GC/epGO_0.8_ and GC/epGO_1.3_ in a solution containing 5 mM K_3_Fe(CN)_6_ and 0.1 M KCl. (**b**), (**c**) EIS Nyquist plots for epGO_0.8_ and epGO_1.3_, respectively. Inset in (**c**) and (**b**): Randles and modified Randles equivalent circuits, respectively, fitting the data.

To assess the charge transfer capability of the epGO coatings, CVs were obtained at different scan rates for the Fe(CN)_6_^−3^/Fe(CN)_6_^−4^ redox couple at the GC electrodes modified with the epGO_0.8_ and epGO_1.3_ films (see Supplementary Fig. S4). The peak potential separations (ΔE_p_) obtained from the CVs at a scan rate of 100 mV/s (Fig. 6a) were 110 and 50 mV for the two coatings, respectively. These values were compared to those reported for pristine graphene (PG), GO, and GO patched with PG sheets (GO/PG) under similar conditions (108, 162 and 56 mV, respectively)^41^. Faster electron transfer can be induced in the epGO coatings obtained at higher applied potentials. The ΔE_p_ value for epGO_1.3_ at a scan rate of 100 mV/s was 50 mV. A lower value (30 mV) was obtained at a low scan rate of 5 mV/s, and this value was lower than the expected theoretical value of 59 mV at 25 °C, suggesting adsorption of the redox species onto the coatings obtained via EP, which was also reported for the GO/PG modified electrodes^41^. The charge transfer resistance (R_CT_) for the epGO films was analyzed using electrochemical impedance spectroscopy (EIS). Two different models fit the EIS data obtained for these films. A modified Randles model with two constant phase elements (CPEs), which has been suggested for non-homogeneous or porous coatings^42^, fits the data for the epGO_0.8_ coating (Fig. 6b). However, the more conventional Randles model, which consists of a CPE as well as a Warburg diffusion (W) element, fits the data obtained from the epGO_1.3_ coating (Fig. 6c) and was also suggested for GO and rGO^43^. This result was attributed to the non-homogeneous nature and incomplete surface coverage that characterize the epGO_0.8_ coatings compared to the coatings obtained at higher applied potential. The R_CT_ for the two coatings was estimated to be 3500 and 39 Ω cm^2^ for epGO_0.8_ and epGO_1.3_, respectively, using the models. The results indicate that while the resistance of the epGO_0.8_ coating is at least one order of magnitude higher than that reported for GO (~197 Ω cm^2^)^43^, the resistance of epGO_1.3_ is similar to that reported for electrochemically reduced GO (~33 Ω cm^2^)^43^. The high conductivity exerted by the coatings obtained at high anodic potentials is consistent with the XPS data, indicating decarboxylation and C=C conjugation (Figs 4c and 5). However, although highly conductive, a thickness of only ≤30 nm could be attained for epGO_1.3_. This finding suggested that unlike the growth of the non-conductive polyphenol coatings, the process for the epGO coating terminates (reduction of CV peak currents, Figs 1a and S1b in Supplementary) due to a lack of accessible phenolic groups on the electrode surface, as indicated by XPS (Fig. 4c).

## Conclusions

A process was demonstrated in which GO sheets were combined at low applied anodic potentials (≤0.8 V) through the ether groups formed via electro-oxidation of the phenolic edge groups. The higher applied potentials (~1.1 V) formed carboxyl-rich coatings, which upon further oxidation at oxygen evolving potentials (~1.3 V), were converted via decarboxylation to coatings with lower O/C ratios and higher content of C=C bonds, which extend the conjugation of the aromatic rings. The coating formation process is considered equivalent to EP of monomeric organic molecules and leads to coatings that are considerably more conductive at oxygen evolving potentials. This finding indicates the possible involvement of oxygen active species in dictating the coating structure. Preliminary experiments conducted using amino-doped GO indicated that it is possible to extend the formation of the coatings to other graphene derivatives through anodic EP. While the previously reported cathodically electrodeposited coatings were obtained due to the less soluble nature of rGO in comparison to GO, the present coatings were based on the formation of chemically bonded GO sheets. The possibility to control the properties (such as thickness and conductivity) of the new coatings by controlling the electrochemical preparation parameters (potential/current density) make them good candidates for possible applications in various electrocatalytic and energy conversion systems.

## Methods

### Materials

Aqueous solutions were prepared with deionized water (18.2 MOhm-cm, Millipore), and all compounds and reagents were of analytical grade. The GO water dispersion (4 mg/mL) and ITO electrodes were obtained from Sigma-Aldrich.

### Preparation of coatings

The electrochemical system for EP was carried out using a three-electrode system immersed in a solution of 2 mg/mL GO in 0.1 M NaHCO_3_. ITO, Au-coated quartz crystal, or GC (geometric areas of 2, 0.21, and 0.07 cm^2^, respectively); Pt wire; and Ag/AgCl/KCl (satd.) were used as the working, counter, and reference electrodes, respectively. In a typical process, the ITO and Au-coated quartz crystal electrodes were first cleaned with acetone and then washed with water. The GC electrodes were polished with alumina and then washed thoroughly with water. The CV and CA experiments were carried out using a Gamry potentiostat (series G™300). Changes in the mass of the coatings obtained via EP (epGO) were monitored using a Gamry EQCM 10 M quartz crystal microbalance (Gamry Instruments) and 10 MHz Au-coated quartz crystals (Gamry Instruments). The CA experiments were performed with the assistance of magnetic stirring. Samples coated with cast GO (cGO) were prepared by dropping 143 µL/cm^2^ of an aqueous suspension of GO (4 mg/mL) on the substrate surface and were dried under room conditions.

### Characterization and measurements

The electrochemical behavior of the epGO coatings was characterized via CV and EIS. The measurements were performed using a Gamry potentiostat with a conventional three-electrode system. The EIS measurements were performed with an open circuit voltage by applying a sinusoidal voltage of 10 mV, and the spectra were recorded in the frequency range from 0.1 Hz to 300 kHz. The EIS 300 software (Gamry) was used for data collection, and the obtained impedance plots were fitted with equivalent circuits provided by Echem Analyst (Gamry) software.

The thickness and surface roughness of the films on the ITO substrate were measured using an atomic force microscope (AFM, Cypher-ES, Asylum Research/Oxford Instruments). To measure the thickness, the films were scratched to expose the substrate, and the AFM was used to scan from the film top to the exposed substrate surface at three different locations for each sample.

The morphology of the film was examined using high resolution scanning electron microscopy (HR-SEM, JEOL JSM-7400F). Transmission electron microscopy (TEM) imaging was carried out using a Tecnai 12 G2 TWIN. The TEM samples were prepared by directly scratching off a portion of the epGO onto a Cu grid coated with lacey carbon.

The chemical nature of the coatings on ITO was examined using X-ray photoelectron spectroscopy (XPS) with an Al X-ray source and monochromator (ESCALAB 250). The attenuated total reflectance Fourier transform infrared (ATR-FTIR) spectra for the coatings on Au were obtained using a Bruker Tensor 27 FTIR with a Bruker Platinum ATR accessory equipped with a single reflection diamond crystal. The Raman spectra on ITO were obtained using the excitation source of a Melles-Griot Argon laser (514.5 nm, LabRam HR).

## Electronic supplementary material

Supplementary information
