# Supplementary material for: Graphene Oxide Sheets Combine into Conductive Coatings by Direct Oxidative Electropolymerization
Source: Sci Rep. 2017 Jul 10;7:4987. doi: 10.1038/s41598-017-05269-1 (PMC5504004; doi:10.1038/s41598-017-05269-1)
Supplement: Supplementary file 1 — Supplementary information [file 41598_2017_5269_MOESM1_ESM.pdf]

Graphene Oxide Sheets Combine into Conductive Coatings by via Direct Oxidative Electropolymerization

S. Halevy[1], Y. Bochlin[1], Y. Kadosh[1], A. Kaplan[2], H. Avraham[3], A. Nissim[3], R. Ben Hamo[1], T. Ohaion-Raz[3], E. Korin[1], A. Bettelheim[1]*

Supplementary Information

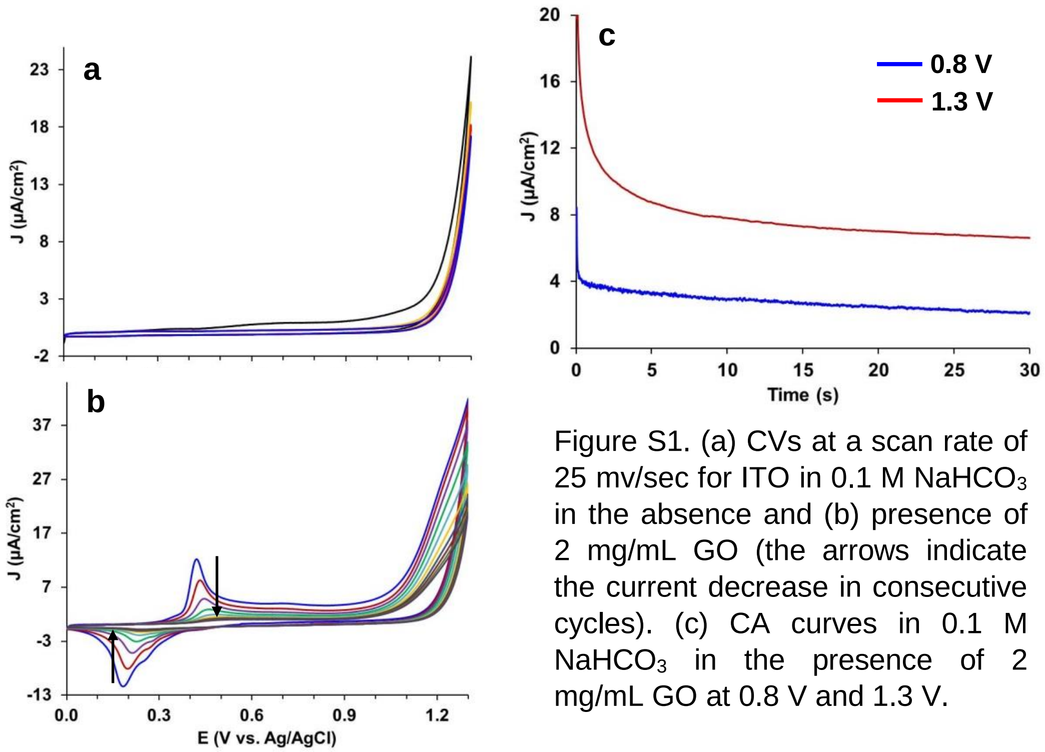

Figure S1. (a) CVs at a scan rate of 25 mv/sec for ITO in 0.1 M $NaHCO_3$ in the absence and (b) presence of 2 mg/mL GO (the arrows indicate the current decrease in consecutive cycles). (c) CA curves in 0.1 M $NaHCO_3$ in the presence of 2 mg/mL GO at 0.8 V and 1.3 V.

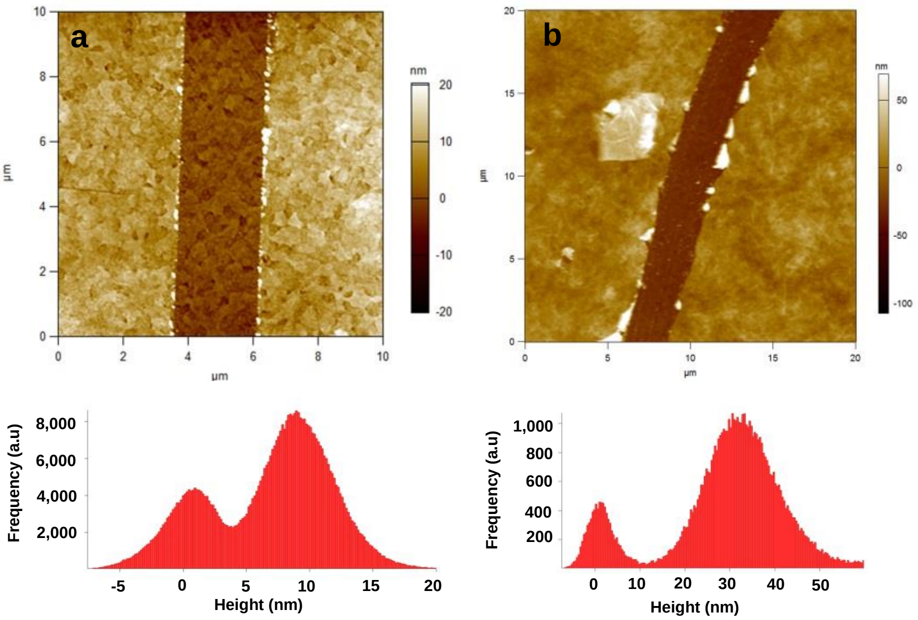

Figure S2. AFM topographic images and the corresponding height histograms of scratched ITO/epGO$_{0.8}$ and ITO/epG$_{1.3}$ (a and b, respectively).

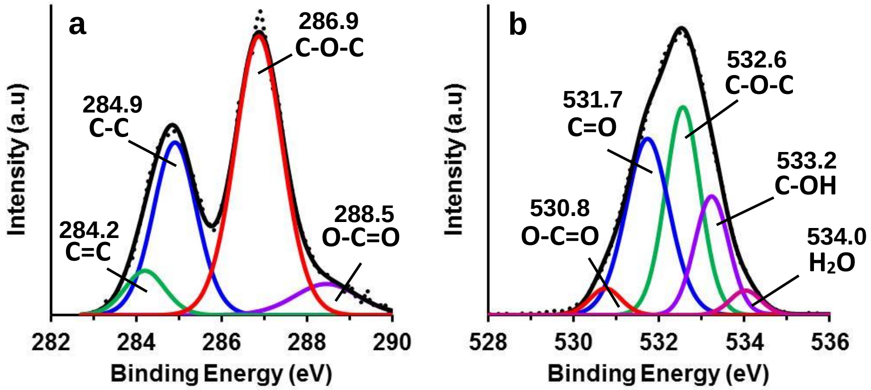

Figure S3. XPS C1s (a) and O1s (b) spectra for ITO/cGO.

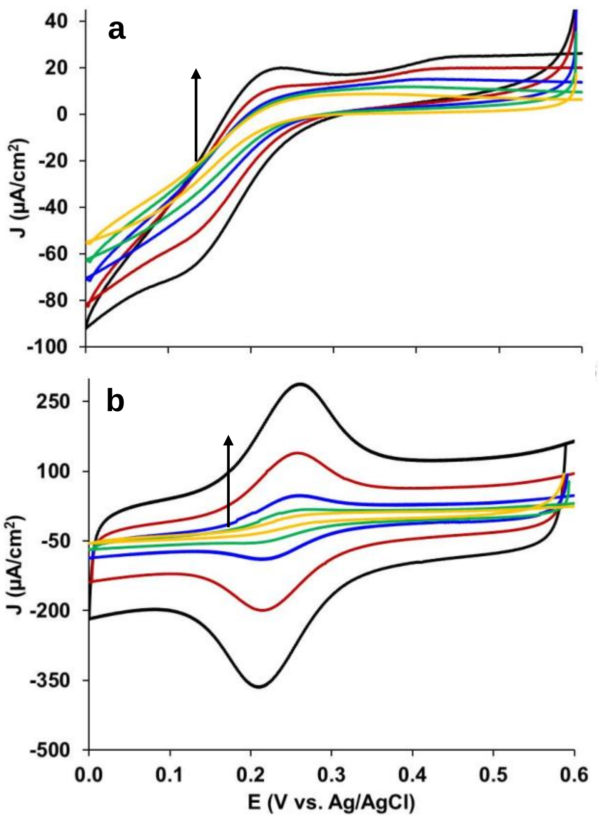

Figure S4. CVs for GC/epGO$_{0.8}$ (a) and GC/epGO$_{1.3}$ (b) in a solution of 5 mM $K_3Fe(CN)_6$ and 0.1 M KCl at scan rates of 5, 10, 20, 50, and 100 mV/s (increasing current peaks at increasing scan rates according to arrows).
